# The Comparison of the Gut Microbiome Composition, Serum Inflammatory Markers and Faecal Short‐Chain Fatty Acids Among Individuals With Type 1 and 2 Diabetes Mellitus With Healthy Controls: A Case–Control Study

**DOI:** 10.1002/edm2.70071

**Published:** 2025-06-17

**Authors:** Hossein Yarmohammadi, Masood Soltanipur, Mahdi Rezaei, Hanieh‐Sadat Ejtahed, Maedeh Raei, Alireza Razavi, Seyed Mohsen Mirhosseini, Mehrangiz Zangeneh, Delaram Doroud, Abolfazl Fateh, Seyedalireza Seyed Siamdoust, Seyed Davar Siadat

**Affiliations:** ^1^ Department of Mycobacteriology and Pulmonary Research Pasteur Institute of Iran Tehran Iran; ^2^ Endocrinology and Metabolism Research Center Endocrinology and Metabolism Clinical Sciences Institute, Tehran University of Medical Sciences Tehran Iran; ^3^ Quality of Life Department Breast Cancer Research Center, Motamed Cancer Institute, ACECR Tehran Iran; ^4^ Health Research Center Chamran Hospital Tehran Iran; ^5^ Gastrointestinal Cancer Research Center Non‐Communicable Diseases Institute, Mazandaran University of Medical Sciences Sari Iran; ^6^ Research Center for Clinical Virology Tehran University of Medical Science Tehran Iran; ^7^ Farabi Eye Hospital, Tehran University of Medical Sciences Tehran Iran; ^8^ Cardiovascular Research Center Shahid Beheshti University of Medical Sciences Tehran Iran; ^9^ Department of Infectious Disease Faculty of Medicine, Tehran University of Medical Sciences, Islamic Azad University Tehran Iran; ^10^ Regulatory Department Production and Research Complex, Pasteur Institute of Iran Tehran Iran; ^11^ Department of Anesthesiology School of Medicine, Iran University of Medical Sciences Tehran Iran; ^12^ Microbiology Research Center (MRC), Pasteur Institute of Iran Tehran Iran

**Keywords:** faecal short‐chain fatty acids (SCFAs), gut microbiota, inflammatory markers, type 1 diabetes mellitus, type 2 diabetes mellitus

## Abstract

**Background:**

This study aimed to compare the gut microbiome (GM) composition, serum inflammatory markers and faecal short‐chain fatty acids among individuals with type 1 and type 2 diabetes mellitus (DM) and healthy controls.

**Methods:**

This case–control study examined 49 subjects with type 2 DM, 21 with type 1 DM and 40 healthy controls. Blood and faecal samples were collected. Serum inflammatory markers, including CRP, IL‐1β, IL‐6, TNF‐α and IFN‐γ, were measured using enzyme‐linked immunosorbent assays (ELISA). Bacterial populations were quantified using RT‐qPCR and NGS. Faecal metabolites were analysed using gas chromatography.

**Results:**

Simpson's alpha diversity was higher among types 1 and 2 DM than in the control. The frequency of the bacterial genera *Gemmiger*, *Dorea*, *Collinsella*, *Escherichia/Shigella*, *Dialister*, *Coprococcus*, *Achromobacter*, *Intestinimonas* and *Allisonella* in type 2 DM was higher than in the control, and the frequency of the genera *Romboutsia* and *Clostridium* was decreased in type 2 DM. The frequency of the *Prevotella*, *Bacteroides* and *Faecalibacterium* genera in type 1 DM was lower than in the other groups. Acetate, propionate and butyrate levels were significantly higher in type 2 DM patients compared to the other groups. Participants with diabetes had significantly higher hs‐CRP, IL1‐β, TNF, IL‐6 and IFG levels compared to the controls. Compared to healthy controls, both T1DM and T2DM patients showed a significant increase in the abundance of the *Lactobacillus* genus (*p* = 0.01) and a decrease in *Faecalibacterium* (*p* = 0.02). Additionally, serum levels of IL‐6 and TNF‐α were significantly elevated in T2DM patients (*p* = 0.003 and *p* = 0.005, respectively). Faecal levels of butyrate were significantly reduced in both diabetic groups compared to the controls (*p* = 0.004).

**Conclusion:**

By determining the GM alterations in patients with diabetes, interventional strategies could be designed to modulate the GM composition as an adjunctive therapy in diabetes.

## Graphical abstract

Gut microbiome, inflammation and short‐chain fatty acids in type 1 and type 2 diabetes.

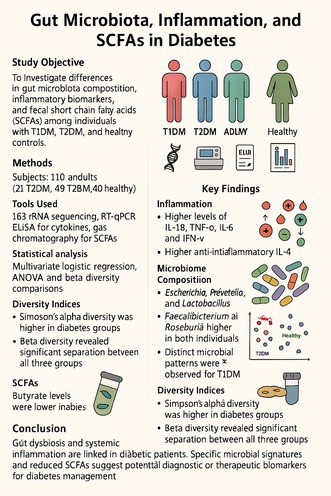

**Abbreviations**
DM: diabetes mellitus
FBS: fasting blood sugar
g: gram
GM: gut microbiota
HDL: high‐density lipoprotein cholesterol
hs‐CRP: high‐sensitivity C‐reactive protein
IFG: interferon‐gamma
IL1‐β: interleukin‐1‐beta
IL‐4: interleukin‐4
IL‐6: interleukin‐6
LDL: low‐density lipoprotein cholesterol
LPS: lipopolysaccharides
mg: milligrams
mL: millilitre
NGS: next‐generation sequencing
NIMAD: national institute for medical research development
RT‐qPCR: real‐time quantitative polymerase chain reaction
SCFAs: short‐chain fatty acids
TC: total cholesterol
TG: triglycerides
TNF: tumour necrosis factor
VLDL: very low‐density lipoprotein cholesterol
μl: microliter

## 1 Introduction

Diabetes mellitus (DM) is among the most prevalent metabolic disorders globally, characterised by elevated blood sugar levels and glucose intolerance. This condition is divided into two categories: type 1 and type 2. Both genetic and environmental factors, such as sedentary habits and high‐calorie diets, contribute to the risk of developing diabetes [1]. Recent research has highlighted the gut microbiota (GM) as a significant factor in the development of diabetes [2]. The GM consists of diverse symbiotic microorganisms residing in the intestine, forming a dynamic ecosystem within the host. The composition and functionality of the GM are influenced by the host's diet, genetics and other lifestyle factors [3, 4, 5]. Interactions between the host and its microbiota impact chronic diseases and various metabolic pathways, including the production of bile acids, short‐chain fatty acids (SCFAs), biogenic amines and xenometabolites [6], [7].

Chronic inflammation is suggested as a key factor in diabetes. The GM is a rich source of components, such as lipopolysaccharides (LPS), which interact with host immune cells, binding to immune receptors and triggering a pro‐inflammatory response [8]. As a result, LPS and peptidoglycans from the GM play roles in initiating and progressing inflammation in peripheral tissues, leading to metabolic endotoxemia and associated diseases [9]. An increase in non‐lactic acid bacteria may inhibit mucin synthesis, potentially causing β cell autoimmunity and resulting in type 1 DM [10]. In type 1 DM, immune system dysregulation, including T‐cell infiltration, has been shown to contribute to organ damage, such as glomerular structural changes in the kidneys and may reflect systemic inflammation driven by GM dysbiosis [11]. Additionally, the GM's involvement in crucial regulatory pathways like insulin signalling and glucose homeostasis underscores its connection with type 2 DM [12]. GM dysbiosis has been shown to contribute to chronic inflammation and insulin resistance in type 2 DM, highlighting the role of GM alterations in inflammatory responses [13].

To manage blood sugar effectively, a deeper understanding of GM changes in DM patients is crucial. This study aims to explore GM patterns and related metabolites in individuals with type 1 and type 2 DM.

## 2 Methods

### 2.1 Study Design, Participants and Sampling

In this case–control study, 110 adult subjects were enrolled. This study comprised 49 individuals with type 2 DM, 21 with type 1 DM and 40 healthy non‐diabetic subjects. The ages of all participants ranged from 20 to 65 years. A power analysis was performed using GPower software (version 3.1.9.7) to determine the minimum required sample size. Based on prior studies evaluating gut microbiota differences in diabetic populations, we calculated that a total sample size of 102 participants (34 per group) would achieve 80% power to detect medium effect sizes (Cohen's d = 0.5) with a significance level of α = 0.05. To ensure statistical robustness and account for potential dropouts, we enrolled 110 participants in total. All research was performed under guidelines approved by the ethical committee of the National Institute for Medical Research Development (NIMAD). All methods of this study were performed under the relevant guidelines and regulations. Informed consent was obtained from all participants. Laboratory parameters and anthropometric measurements were assessed following our previously established protocols [14]. Demographic data were also collected. The exclusion criteria were pregnancy, lactation, smoking and consumption of antibiotics, corticosteroids, probiotics and prebiotics during the 3 months before the study. The fresh faecal samples of participants were collected in sterile cups. The samples were collected by the subjects at home and immediately stored in ice packs then transported within two hours to our facilities. For analysis of the GM, samples were stored at −80°C.

### 2.2 Serological Tests

Following an overnight fast of 12–14 h, blood samples were collected from participants using serum separator tubes (SST II Advance, BD Vacutainer). These tubes contain a clotting activator and a gel separator to facilitate serum isolation. The serum was promptly separated by centrifugation at 1300 g for 10 min at room temperature and subsequently stored at −80°C until analysis. Concentrations of fasting blood sugar (FBS), total cholesterol (TC), triglycerides (TG), high‐density lipoprotein cholesterol (HDL), low‐density lipoprotein cholesterol (LDL), very low‐density lipoprotein cholesterol (VLDL) and high‐sensitivity C‐reactive protein (hs‐CRP) were quantified using Roche kits with an auto‐analyser (Hitachi, Cobas C 311, Roche Diagnostics GmbH). Serum insulin levels were determined using an enzyme immunoassay kit (Monobind Inc., Lake Forest, CA, USA). The results of all biochemical and inflammatory parameters were expressed in appropriate units. The reference ranges and clinical cut‐off values were as follows: FBS: mg/dL, reference range: 70–99 mg/dL, diagnostic cut‐off for diabetes: ≥ 126 mg/dL; TC: mg/dL, reference: < 200 mg/dL; TG: mg/dL, reference: < 150 mg/dL; HDL‐C: mg/dL, desirable: >40 mg/dL (men), > 50 mg/dL (women); LDL‐C: mg/dL, optimal: < 100 mg/dL; VLDL‐C: mg/dL, reference: 5–40 mg/dL; hs‐CRP: mg/L, low risk: < 1.0 mg/L, moderate risk: 1.0–3.0 mg/L, high risk: > 3.0 mg/L; Insulin: μIU/mL, reference: 2.6–24.9 μIU/mL; IL‐6: pg/mL, reference: < 7 pg/mL; TNF‐α: pg/mL, reference: < 8 pg/mL.

The inflammatory markers, including hs‐CRP, interleukin‐1‐beta (IL‐1β), tumour necrosis factor‐alpha (TNF‐α), interleukin‐6 (IL‐6) and interferon‐gamma (IFN‐γ), were selected due to their well‐established roles in mediating chronic low‐grade inflammation associated with DM. Dysbiosis of the gut microbiota can disrupt intestinal barrier integrity, leading to increased translocation of microbial products and activation of immune responses, which in turn elevates these inflammatory markers. Measuring these biomarkers allows for an in‐depth evaluation of the connection between gut microbiota alterations and systemic inflammation in patients with diabetes. The levels of cytokines IL1‐β, TNF‐ α, IL‐6, IFN‐γ and interleukin‐4 (IL‐4), as inflammatory markers, were measured and compared between the diabetic and control groups using ELISA kits (Abnova, Taipei, Taiwan).

### 2.3 DNA Isolation and Quantitation

DNA was extracted from approximately 200 mg of stool samples using the QIAamp DNA Stool Mini Kit (QIAGEN, GmbH, Germany) following the manufacturer's protocol [15]. Stool samples were homogenised in the ASL buffer provided with the kit to ensure bacterial cell lysis and the removal of PCR inhibitors. After lysis, the samples were heated at 95°C for 5 min, followed by centrifugation to pellet debris. The supernatant was then processed through silica membrane spin columns for DNA binding, washing and elution. The concentration and purity of the extracted DNA were measured using a NanoDrop spectrophotometer (Thermo Scientific NanoDrop, USA) and stored at −80°C until further analysis. Absorbance was recorded at 260 nm for DNA quantification, while the A260/A280 ratio was used to assess protein contamination and the A260/A230 ratio was used to detect contaminants such as carbohydrates and phenols. DNA concentrations were reported in ng/μL. For example, a typical sample had a DNA concentration of 50 ng/μL, with purity ratios of A260/A280 = 1.9 and A260/A230 = 2.1, indicating high‐quality DNA suitable for downstream analysis.

### 2.4 Sequencing Using Real‐Time Quantitative Polymerase Chain Reaction (RT‐qPCR)

Real‐time quantitative polymerase chain reaction (RT‐qPCR) was employed to quantify the abundance of eight specific bacterial populations. The qRT‐PCR analysis targeted the 16S rRNA gene of eight specific bacterial genera: *Escherichia, Prevotella, Lactobacillus, Bifidobacterium, Akkermansia, Roseburia, Faecalibacterium and Bacteroides*. The 16S rRNA gene was selected due to its conserved and hypervariable regions, enabling accurate identification of bacterial taxa. These genera were chosen based on their established associations with gut health, inflammation and diabetes‐related metabolic disturbances. For instance, butyrate‐producing genera like *Roseburia* and *Faecalibacterium* are linked to anti‐inflammatory effects, whereas *Escherichia and Prevotella* have been implicated in promoting inflammation and metabolic dysfunction in diabetes. Specific primers for the 16S ribosomal RNA (rRNA) gene of bacteria were selected as listed in the Supporting Information S1. To assess the quality and specificity of the primers, basic local alignment search tool (BLAST) searches were conducted on the National Center for Biotechnology Information (NCBI) website. RT‐qPCR was carried out using the LightCycler 96 system by Roche (Switzerland). All experiments were conducted in duplicate. To quantify bacterial concentration, standard curves were constructed using serial 10‐fold dilutions of known concentrations of genomic DNA of standard *Escherichia coli* (ATCC 25922). Subsequently, quantitative analysis of bacterial copy numbers for eight targeted genera was performed using one gram (g) of faecal samples.

### 2.5 16S Library Preparation and Next‐Generation Sequencing (NGS)

To sequence the 16S rRNA using NGS, we employed the Power Microbiome RNA isolation kit (MOBIO Laboratories Inc., Germany) for DNA extraction. Bacterial genome sequencing was conducted on the Illumina MiSeq platform (Nucleomics core, KU Leuven). Raw sequencing data were processed using the DADA2 pipeline (version X.X) in R. This pipeline included quality filtering, trimming of low‐quality bases, removal of chimeric sequences and merging of paired‐end reads. Adapter sequences were removed during preprocessing. Quality filtering parameters were set to truncate reads at positions where quality scores dropped below 20, and sequences shorter than 200 bp were discarded. This comprehensive approach ensured high‐resolution identification of amplicon sequence variants (ASVs) and minimised sequencing errors. Additionally, taxonomic classification was performed using the Ribosomal Database Project (RDP) classifier (version 2.12).

The initial preprocessing of 16S sequences was conducted using the LotuS and Deficiency of Adenosine Deaminase 2 (DADA2) pipelines with default parameters. The classification was performed using the RDP classifier v 2.12. Sequences containing chloroplasts, mitochondrial families and unclassified bacteria were excluded. The remaining sequences were normalised to ten thousand reads per sample. The alpha and beta diversity indices were computed using the vegan and phyloseq R packages. Alpha diversity of the microbiome was measured based on operational taxonomic units (OTUs) using the Richness and Simpson indices. The Richness index quantifies the variety of microorganisms in the gut, while the Simpson index takes into account both the number and relative abundance of bacterial species. Beta diversity was visualised at the genus level using the Bray–Curtis dissimilarity index and the principal coordinates analysis (PCoA) method. Subsequent analyses were performed at the genus level. Enterotyping was conducted using the Dirichlet multinomial mixtures (DMM) method in R software. To validate the enterotyping, the samples from the current study were compared with a comprehensive Belgian population study comprising 1106 samples.

### 2.6 Quantification of Faecal Metabolites

SCFAs in the faeces were quantified using gas chromatography. Approximately 100 mg (mg) of faecal sample were suspended in one millilitre (mL) of saturated 36% Sodium chloride (NaCl) solution. An internal standard (50 μL (μl) of 10.7 μm (μM) 2‐ethyl butyric acid by Merck, Munich, Germany in Milli‐Q (MQ) water) was added, and the samples were homogenised using glass homogeniser beads. After the addition of 150 μL of 96% Sulphuric acid (H_2_SO_4_), SCFAs were extracted in 3 mL of ether. The ether layer was collected and dried with 150 mg of Na_2_SO_4_. The supernatant (0.5 μL) was analysed using the gas chromatography—flame ionisation detection (GC—FID) method (Agilent). The resulting chromatograms were processed using Agilent Technologies ChemStation. Acetate, propionate and butyrate were quantified using appropriate standard curves.

### 2.7 Statistical Analyses

Data analysis was performed using Statistical Package for Social Sciences (SPSS) software version 16 and R software version 3.5.0. A *p*‐value of less than 0.05 was considered statistically significant. The normality of data distribution was evaluated using the Kolmogorov–Smirnov test and by examining histogram plots. Logarithmic transformation was applied to variables that did not have a normal distribution, and if they still did not achieve a normal distribution, non‐parametric tests were used. The chi‐square test was employed to examine the equality in the distribution of men and women across the three groups. The three groups were statistically compared using the one‐way analysis of variance for variables with a normal distribution, and the Kruskal–Wallis test was utilised for variables that exhibited a non‐normal distribution. After the one‐way analysis of variance, the Bonferroni test was conducted for multiple comparisons. To assess the combined effects of diabetes status and age on gut microbiome (GM) composition and pro‐inflammatory cytokines, participants were stratified into three age groups: 20–35 years (young adults), 36–50 years (middle‐aged adults) and 51–65 years (older adults). A two‐way ANOVA was performed to evaluate the interaction between age and diabetes status across bacterial genera and inflammatory markers. Post hoc analyses were conducted where appropriate to further investigate significant interactions. All variables that demonstrated statistical significance in binary analyses were included in a multivariate logistic regression model to identify independent predictors of diabetes status. The model was adjusted for potential confounders such as age, gender and BMI. Results were reported as odds ratios (ORs) with 95% confidence intervals (CIs), and a *p*‐value < 0.05 was considered statistically significant.

## 3 Results

### 3.1 Demographic Characteristics

The average age of participants in type 1 DM, type 2 DM and control group was 35.4 years, 57.2 years and 38.0 years, respectively. The average age of adult patients with type 2 DM was significantly higher compared to people with type 1 DM and healthy people (*p*‐value < 0.001). The three groups had similar sex distributions (*p*‐value > 0.05). The use of DM medications, including metformin and insulin, was observed in 14% and 95% of people with type 1 DM, as well as in 67% and 30% of type 2 DM patients. Additionally, 14% and 28% of type 1 and type 2 DM patients reported using lipid‐lowering statins. In terms of weight and BMI, there was a significant difference between the groups, patients with type 1 DM had less weight than the other two groups (*p*‐value < 0.05).

### 3.2 Serological Findings

Anthropometric indices and biochemical variables of non‐diabetic adults and patients with type 1 and type 2 DM are given in Table 1. Types 1 and 2 DM groups had higher FBS concentrations than the healthy group (*p*‐value < 0.001), but serum insulin concentration was not significantly different between groups (*p*‐value > 0.05). TC and LDL were significantly higher in the healthy control group than in the other groups (*p*‐value < 0.05), and HDL was also significantly higher in the control group (*p*‐value < 0.001). Statistically, there was no significant difference in TG and VLDL between the studied groups (*p*‐value > 0.05). Hs‐CRP concentration was significantly lower in non‐diabetic subjects compared to patients with diabetes (*p*‐value < 0.001).

**TABLE 1:** The anthropometric indices and biochemical variables of non‐diabetic adults and patients with type 1 and type 2 DM.

Variables	Type 1 DM (mean ± SD)	Type 2 DM (mean ± SD)	Healthy controls (mean ± SD)	*p*
	*N* = 21 (M:8/F:13)	*N* = 49 (M:22/F:27)	*N* = 40 (M:10/F:30)	
Age (Years)	35.4 ± 12.4	57.2 ± 9.9	38.0 ± 9.8	**< 0.001**
Weight (kg)	61.7 ± 23.8	75.5 ± 13.6	72.4 ± 15.4	**0.01**
BMI (kg/m^2^)	21.9 ± 6.4	28.0 ± 4.4	26.7 ± 6.2	**< 0.001**
FBS (mg/dL)	182.3 ± 106.7	154.0 ± 51.2	79.3 ± 7.2	**< 0.001**
Insulin (mU/L)	15.3 ± 25.0	13.1 ± 12.5	11.0 ± 5.8	0.52
TC (mg/dL)	154.1 ± 41.7	151.7 ± 43.9	194.0 ± 40.6	**< 0.001**
TG (mg/dL)	111.98 ± 63.9	147.9 ± 94.2	116.5 ± 48.6	0.07
HDL (mg/dL)	43.2 ± 6.5	39.3 ± 8.0	46.6 ± 9.0	**< 0.001**
LDL (mg/dL)	88.5 ± 32.4	82.8 ± 32.2	100.7 ± 23.8	**0.02**
VLDL (mg/dL)	22.4 ± 12.8	26.4 ± 11.8	23.3 ± 9.7	0.3
hs‐CRP (mg/dL)	15.5 ± 1.8	11.5 ± 2.5	4.5 ± 3.1	**< 0.001**

Abbreviations: BMI, body mass index; DM, diabetes mellitus; F, female; FBS, fasting blood sugar; HDL, high‐density lipoprotein cholesterol; hs‐CRP, high‐sensitivity C‐reactive protein; LDL, low‐density lipoprotein cholesterol; M, male; N, number; SD, standard deviation; TC, total cholesterol; TG, triglycerides; VLDL, very low‐density lipoprotein cholesterol.

**FIGURE 1:**
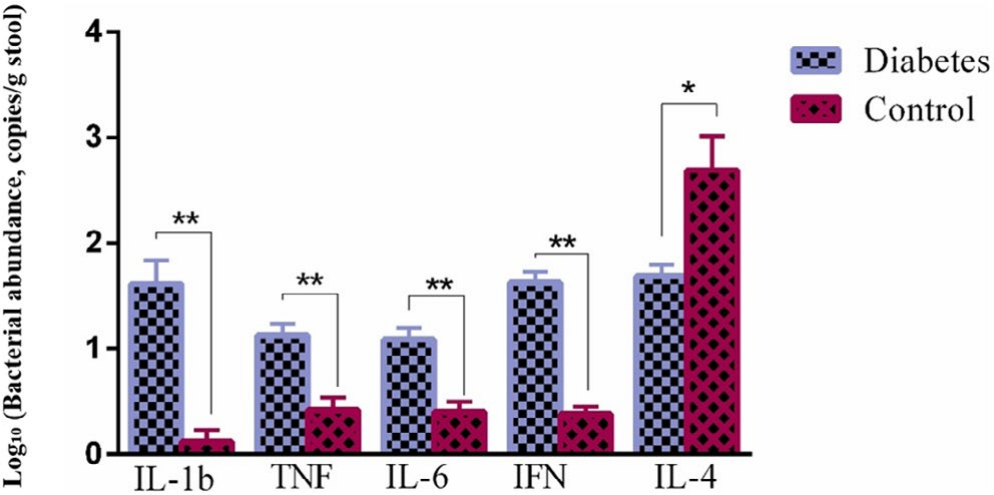
Log‐transformed relative abundance (Log_10_) of key bacterial genera in stool samples from type 1 diabetes, type 2 diabetes and healthy control groups. Abundance is expressed in copies per gram of stool (copies/g).

In the measurement of inflammatory factors by ELISA method, it was found that the level of cytokines IL1‐β, TNF, IL‐6 and IFG was significantly higher in patients with diabetes (*p*‐value < 0.01) and the level of IL‐4 was significantly higher (*p*‐value < 0.05) among healthy people as is shown Figure 1.

### 3.3 GM Composition

Following quality control and taxonomic classification, a total of 250 distinct bacterial genera were identified across all samples. This comprehensive profiling allowed for an in‐depth comparison of the GM composition among T1DM, T2DM and healthy controls. The Richness α‐diversity index was not significantly different between individuals with diabetes and healthy participants, but Simpson's alpha diversity index was higher in patients than in the control group (*p*‐value =0.02). In terms of the beta biodiversity index, both types 1 and 2 DM and healthy groups significantly differed from each other (*p*‐value =0.02).

**FIGURE 2:**
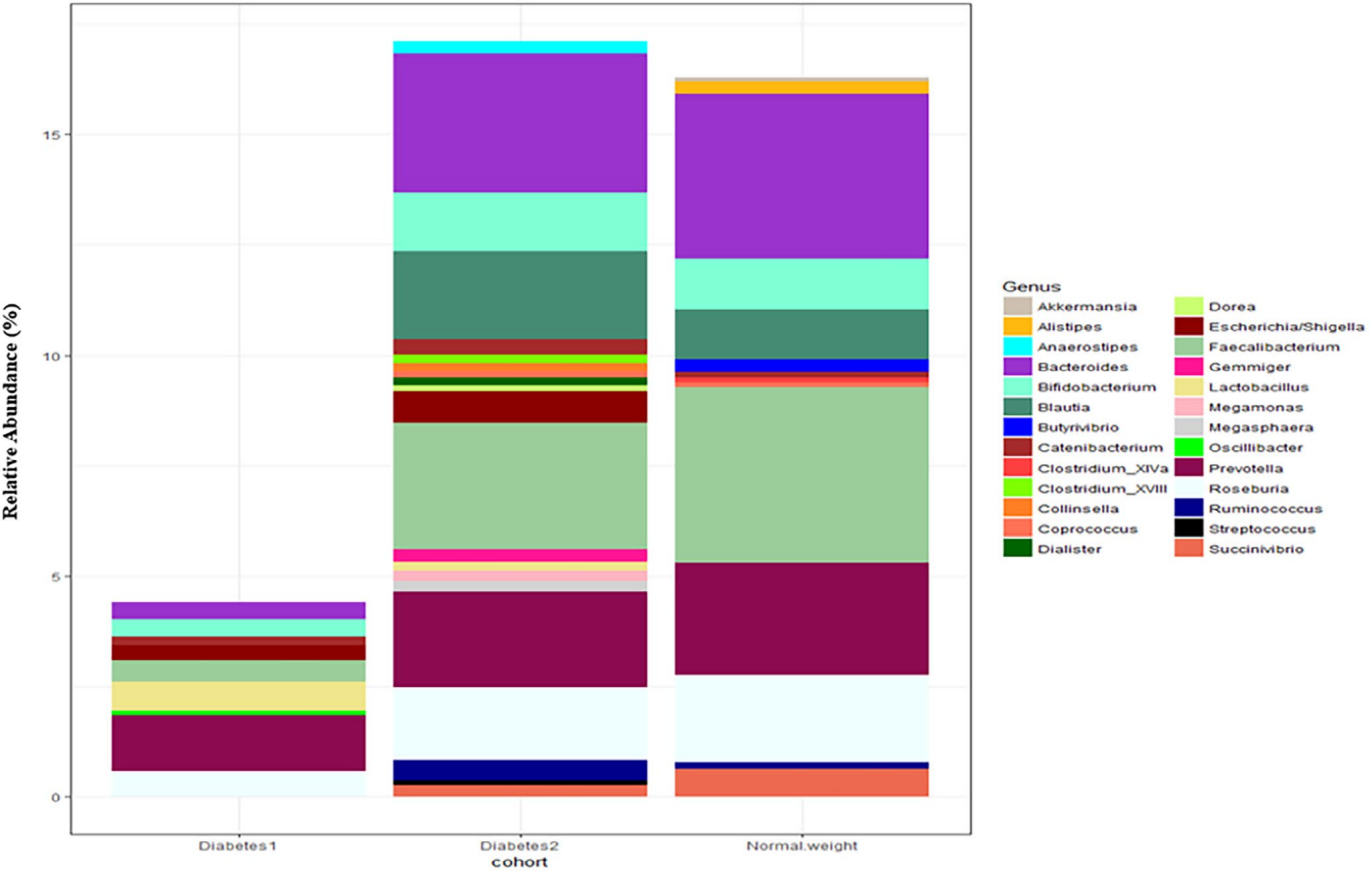
Relative abundance (%) of dominant bacterial genera in stool samples from type 1 diabetes, type 2 diabetes and healthy control groups.

**FIGURE 3:**
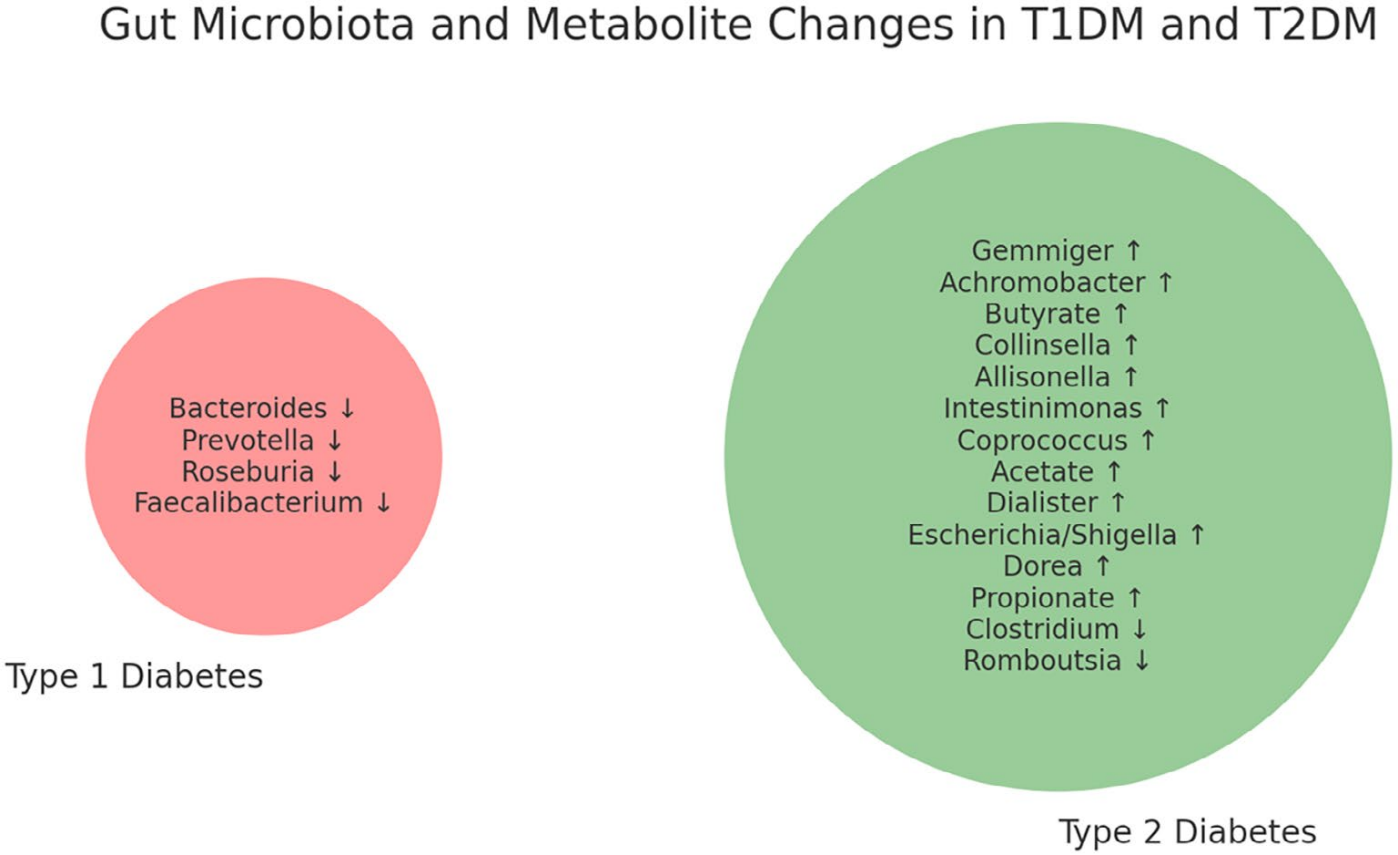
The Venn diagram includes ↑ (increase) and ↓ (decrease) indicators for each bacterial genus and metabolite in T1DM and T2DM.

The frequency of the bacterial genera *Gemmiger*, *Dorea*, *Collinsella*, *Escherichia/Shigella*, *Dialister*, *Coprococcus*, *Achromobacter*, *Intestinimonas*, *Allisonella* in the group of type 2 DM patients was higher than in the healthy group, and the frequency of the genera *Romboutsia* and *Clostridium* was decreased in patients with type 2 DM (*p*‐value < 0.05). In addition, the frequency of *Prevotella*, *Bacteroides* and *Faecalibacterium* genera in individuals with type 1 DM was lower than in the other two groups (*p*‐value < 0.05). The comparison of the GM in DM patients and healthy people is shown in Figure 2. To better illustrate the overlapping and distinct bacterial genera and faecal metabolites between T1DM and T2DM, a Venn diagram was created (Figure 3). This diagram highlights the bacterial taxa and metabolites unique to each condition, providing a clearer understanding of the gut microbiota alterations associated with each disease. Based on the data provided, no bacterial genera were reported as being altered in the same direction in both T1DM and T2DM.

In the *Proteobacteria* phylum, the frequency of *Escherichia genus* in stool samples of healthy controls was significantly lower compared to patients with diabetes (*p*‐value < 0.001). In the *Bacteroidetes* phylum, in type 1 and type 2 DM patients compared to the healthy controls, the frequency of *Prevotella* bacteria was significantly higher and the amount of *Bacteroides genus* was significantly lower (*p*‐value < 0.001). *Roseburia*, a genus of butyrate‐producing bacteria, was significantly increased in healthy controls (*p*‐value =0.02). *Faecalibacterium*, another genus of butyrate‐producing bacteria from the *Firmicutes* phylum, was significantly less in type 1 DM patients compared to the other two groups (*p*‐value < 0.001). In patients with diabetes, the abundance of *Lactobacillus bacteria* compared to the healthy control group significantly increased (*p*‐value < 0.001). No significant difference was observed in the *Akkermansia* level among the three groups (*p*‐value > 0.05). In the investigation of *Actinobacteria*, the abundance of *Bifidobacterium* was significantly higher in healthy controls (*p*‐value =0.04). The bacterial genus abundance of stool samples in patients with diabetes and healthy controls is shown in Table 2.

**TABLE 2:** Relative abundance (%) of bacterial genera among type 1 diabetes, type 2 diabetes and healthy control groups.

Bacterial genus	Type 1 DM (mean ± SD)	Type 2 DM (mean ± SD)	Healthy controls	*p*
	*N* = 21 (M:8/F:13)	*N* = 49 (M:22/F:27)	*N* = 40 (M:10/F:30)	
*Escherichia*	6.0 ± 0.9	5.9 ± 1.0	5.0 ± 1.1	**< 0.001**
*Prevotella*	7.6 ± 0.1	7.6 ± 0.1	6.4 ± 2.0	**< 0.001**
*Lactobacillus*	6.8 ± 0.4	6.8 ± 0.4	4.4 ± 0.9	**< 0.001**
*Bifidobacterium*	5.7 ± 0.9	5.5 ± 0.8	6.0 ± 1.0	**0.04**
*Akkermansia*	4.4 ± 1.9	4.2 ± 2.0	5.0 ± 1.7	0.14
*Roseburia*	5.8 ± 0.8	5.7 ± 1.1	6.4 ± 1.0	**0.02**
*Faecalibacterium*	6.3 ± 0.5	7.3 ± 0.2	7.2 ± 0.8	**< 0.001**
*Bacteroides*	7.6 ± 0.8	7.8 ± 0.1	8.2 ± 0.6	**< 0.001**

*Note:* Abundance values are expressed as relative abundance (%), calculated as the proportion of each bacterial genus relative to the total bacterial community.

Abbreviations: DM, diabetes mellitus; F, female; M, male; N, number; SD, standard deviation.

### 3.4 Faecal Metabolite Concentrations

**FIGURE 4:**
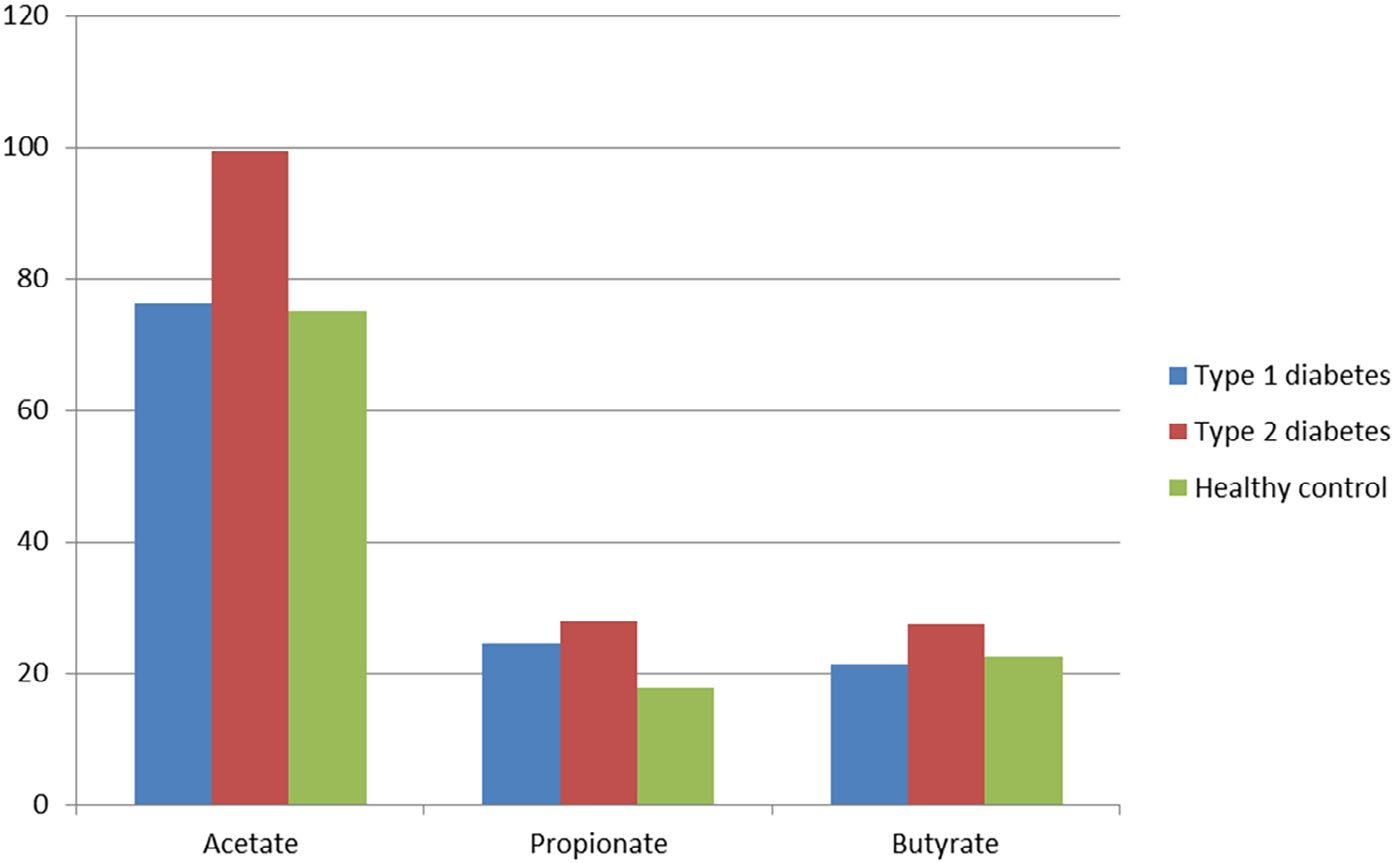
The comparison of faecal metabolite concentration (μmol/g stool) in diabetic patients and healthy people.

The concentration of SFCAs in faeces was compared between three groups and it was found that the concentration of acetate was significantly higher in patients with type 2 DM than in the other two groups (*p*‐value < 0.05). The concentration of propionate in healthy individuals was lower than that of diabetic patients, and the concentration of butyrate in faeces was also higher in patients with type 2 DM than in the other two groups (*p*‐value < 0.05). The comparison of faecal metabolite concentration in patients with diabetes and healthy controls is shown in Figure 4.

## 4 Discussion

We expanded upon two previous pilot studies by utilising the quantitative RT‐qPCR and NGS to characterise the GM composition in patients with type 1 and type 2 DM and healthy adults from NIMAD projects [14]. We observed in individuals with type 2 DM, the abundance of bacterial genera such as *Gemmiger*, *Dorea*, *Collinsella*, *Escherichia/Shigella*, *Dialister*, *Coprococcus*, *Achromobacter*, *Intestinimonas* and *Allisonella* was higher compared to the healthy group. Conversely, the presence of *Romboutsia* and *Clostridium* genera was reduced in patients with type 2 DM. Additionally, individuals with type 1 DM exhibited lower frequencies of the *Prevotella*, *Bacteroides* and *Faecalibacterium* genera when compared to the other two groups. It can be justified that diabetes affects the GM, leading to changes in microbial populations. Factors such as diet, inflammation and metabolic disturbances contribute to this alteration [16]. Also, chronic low‐grade inflammation associated with diabetes may favour the growth of certain bacterial species [17]. It should not be neglected that hyperglycemia and insulin resistance impact nutrient availability, influencing microbial diversity [18]. The GM, including *Akkermansia muciniphila* , *Faecalibacterium prausnitzii* , *Bifidobacterium*, *Lactobacillus*, *E. coli* and *Bacteroides fragilis* , is believed to play a role in regulating metabolic profiles and the immune system. Additionally, it may contribute to chronic, low‐grade inflammation, leading to insulin resistance and type 2 DM [19], [20]. The widespread use of insulin and statins may have influenced the gut microbiota composition, faecal SCFA levels and inflammatory markers observed in this study. Insulin therapy, by improving glycemic control, may reduce gut dysbiosis and intestinal permeability, thereby modulating systemic inflammation. However, some evidence suggests that insulin may alter gut bacterial diversity, indirectly impacting immune responses [21]. Similarly, statins have been reported to modify gut microbial composition by increasing beneficial SCFA‐producing bacteria and reducing pro‐inflammatory taxa [22]. These effects could partly explain the differences in gut microbiota and inflammatory markers observed between diabetic patients and healthy controls. Future studies should control medication use to better isolate the microbiome's role in diabetes pathophysiology.

This study demonstrated that individuals with type 1 DM exhibited a significantly lower abundance of the *Prevotella* and *Bacteroides* genera compared to the other two groups. Furthermore, previous studies on the bacterial abundance of *Prevotella* and *Bacteroides* in type 1 and type 2 DM have concluded inconsistent results [23, 24, 25]. *Prevotella* exhibits varying effects on blood sugar regulation. Specifically, this mucin‐degrading bacterium may compromise intestinal integrity, while simultaneously functioning as a succinate producer. This production enhances intestinal gluconeogenesis, which in turn can inhibit hepatic glucose release [24]. It is essential to recognise that long‐term dietary habits significantly influence the compositions of *Prevotella* and *Bacteroides* in the GM. Research has demonstrated that *Bacteroides* correlate with diets high in protein and animal fats, whereas *Prevotella* is associated with diets abundant in carbohydrates and dietary fibre [26], [27].

As a vital butyrate‐producing genus, *Roseburia* plays a crucial role in promoting gut health. It helps prevent the expression of pro‐inflammatory cytokines in the mucus and effectively modulates the immune system. Studies indicate that higher levels of intestinal *Roseburia* are negatively correlated with plasma glucose, underscoring its potential importance in maintaining glucose homeostasis. Consuming foods that support *Roseburia* can enhance not only gut health but overall metabolic well‐being [28]. Our results also showed that the concentration of *Roseburia* in the faeces of patients with diabetes was lower. Our findings also indicated that the frequency of *Akkermansia* was lower in types 1 and 2 DM groups compared to healthy individuals; however, this difference did not reach statistical significance. In a study employing NGS, Zhang et al. proposed that a reduced abundance of *Akkermansia muciniphila* could serve as a potential biomarker for glucose intolerance [29]. A decrease in the levels of bacteria that produce SCFAs, including *Akkermansia*, *Roseburia* and *Faecalibacterium*, is frequently observed in the intestinal microbiota of patients with diabetes. This reduction is associated with heightened intestinal permeability, which can result in metabolic endotoxemia by permitting pro‐inflammatory substances to pass from the intestinal lumen into the bloodstream [10], [30]. Recent studies examining GM in patients with diabetes, particularly the work by Qin et al. [31] and Karlsson et al. [32], have consistently found reduced levels of butyrate‐producing bacteria such as *Roseburia* and *Faecalibacterium*. Interestingly, we clearly showed that in healthy controls, the genus *Roseburia* showed a significant increase. However, in type 1 DM patients, the genus *Faecalibacterium*, another butyrate‐producing bacterium from the *Firmicutes* phylum, was significantly lower compared to the other two groups.

Consistent with our findings, other studies have shown that *Escherichia*, a genus within the *Proteobacteria phylum*, is more prevalent in patients with type 2 DM [31]. Maskarinec et al.'s study found, similar to ours, an increase in *Escherichia/Shigella* and a decrease in *Clostridium* in type 2 DM patients [33]. Additionally, Thingholm et al.'s research also observed a rise in *Escherichia/Shigella* in type 2 DM [34]. LPS from the outer membrane of gram‐negative bacteria bind to toll‐like receptor 4 (TLR4), triggering the release of pro‐inflammatory cytokines and leading to insulin resistance. Additionally, a higher prevalence of pro‐inflammatory bacteria like *Escherichia* can weaken epithelial integrity, promote chronic inflammation and autoimmune responses and increase the risk of type 1 DM [24].

According to Sedighi et al., type 2 DM is associated with *Lactobacillus* and *Bifidobacterium* frequency in the Iranian populations. *Lactobacillus* was found to be prevalent at significantly higher levels in patients with type 2 DM than in healthy individuals. Conversely, patients with type 2 DM had lower *Bifidobacterium* frequencies than healthy individuals [35]. Comparatively, Le et al. [36] and Halawa et al. [37] reported significantly lower *Lactobacillus* levels in stool samples of type 2 DM patients. Our study confirmed this finding that the number of *Lactobacillus* bacteria significantly increased in DM compared to healthy controls. The observed increase in the abundance of the *Lactobacillus* genus in both T1DM and T2DM patients compared to healthy controls may be attributed to several factors. One plausible explanation is the hyperglycemic state in patients with diabetes, which increases luminal glucose availability, providing a favourable substrate for *Lactobacillus* growth [38]. Additionally, diabetic‐induced changes in gut pH and immune responses may further support the proliferation of Lactobacillus. While certain Lactobacillus strains are beneficial, others have been associated with pro‐inflammatory effects, which could exacerbate metabolic disturbances and inflammation in diabetes [39]. Further studies are needed to distinguish between beneficial and potentially harmful *Lactobacillus* strains in the diabetic GM. *Bifidobacterium* numbers were significantly higher in healthy controls in the *Actinobacteria* investigation as well. In this study, we found that the GM composition of the group was influenced by geography, climate, lifestyle, food, race and culture, making it an important population to study since several factors are known to affect GM composition.

Additionally, some other bacterial genera were altered in faeces samples of patients with diabetes in our report. While no studies have previously linked the *Achromobacter* genus to DM, our study found it to be more prevalent in type 2 DM patients, warranting further investigation. Moreover, although *Romboutsia* hasn't been detected in stool microbiome studies, past research showed a reduction of this bacterium in the mesenteric adipose tissue of type 2 DM patients compared to healthy individuals [40]. Our study found this bacterial genus to be less common in type 2 DM patients. Moreover, *Dialister* and *Clostridium* showed changes in abundance between groups. Naderpoor et al.'s research discovered that higher levels of *Dialister* were linked to reduced insulin sensitivity and increased insulin secretion in type 2 DM patients, and it was inversely related to *Clostridium* levels [41]. This supports the increase in *Dialister* and the decrease in *Clostridium* observed in our study of type 2 DM patients. In the research by Maffeis et al., the frequency of *Dialister* was linked to an increased risk of type 1 DM and autoimmunity [42]. Similarly, Qi et al. noted a decrease in *Dialister* in type 1 DM patients compared to healthy individuals [43]. However, our study did not find a significant difference, likely due to the small sample size for type 1 DM cases.

Our study suggested that acetate metabolism might be altered in DM. Moreover, propionate is involved in various metabolic processes, and its lower concentration in healthy individuals could be relevant. Also, we elucidated that butyrate is known to play a role in gut health and metabolism. These findings are part of a broader scientific landscape, and ongoing research continues to deepen our understanding. In the present study, we concluded that in DM patients, cytokines IL1‐B, TNF, IL‐6 and IFG were significantly elevated, while IL‐4 levels were higher in healthy individuals. Additionally, IL‐4 and IL‐6, as well as IL‐4 and TNF levels, showed significant differences among diabetic subjects. Cytokines play essential roles in coordinating intricate interactions between pancreatic β cells and immune cells during the development of type 1 DM. As a result, they represent potential targets for immunotherapy in this condition [44]. This research suggests that cytokine levels play a crucial role in diabetes. The elevated levels of certain cytokines in diabetic patients may contribute to the disease pathogenesis. Further investigation is needed to understand the underlying mechanisms and potential therapeutic implications.

Researchers have found that the GM composition in type 1 and type 2 DM is heterogeneous in various ways, including race, geography, medical history, lifestyle, dietary habits, study design and GM assessment methods. Our study attempted to eliminate the effects of some confounding factors, such as antibiotics, prebiotics, probiotics and other chronic disorders, by applying some exclusion criteria. However, in our study, we aimed to mitigate the impact of certain confounding variables, such as antibiotics, prebiotics, probiotics and chronic disorders, by excluding participants who exhibited these factors. However, due to the lack of matching across three groups in terms of sex, age and BMI, we were unable to fully eliminate the potential confounding effects of these variables. Furthermore, in a recent investigation, we demonstrated changes in the composition of GM among DM patients. However, due to the study's design limitations, we were unable to definitively establish a causal connection between microbiome alterations and disease. To address this, additional longitudinal studies are necessary to clarify the cause‐and‐effect relationships. Moreover, our investigation focused solely on a restricted set of bacterial genera within the GM. However, a metagenomic analysis has the potential to offer a comprehensive perspective on all microorganisms inhabiting the gastrointestinal tract. Additionally, we acknowledge that the relatively small sample size, particularly in the T1DM group, may limit the generalisability of our findings. However, this study was designed as an exploratory analysis, and stringent inclusion criteria were applied to reduce confounding variables. Future studies with larger, more diverse cohorts are needed to confirm these results and further investigate the role of gut microbiota in diabetes pathogenesis.

## 5 Conclusion

Significant differences exist in the GM and related metabolites between type 1 and type 2 DM patients compared to healthy individuals. In diabetic patients, bacteria‐producing SFCAs were less frequent, while bacterial genera that stimulate the immune system were more prevalent. This study utilised the NGS method for a more accurate and comprehensive measurement of bacterial genera, which is now a common approach for examining GM patterns. The study suggests that analysing GM can aid in diagnosing and identifying the causes of DM and adjusting its composition may benefit diabetes management or treatment.

## Author Contributions

H.Y., M.S. and M.R. performed the experiments, sampling, DNA extraction, 16S rRNA sequencing, SCFAs chromatography, biochemical test, data analysis, prepared figures and tables and contributed to writing the drafts of the paper; H.‐S.E., A.R. and M.R. contributed to writing the drafts of the paper; S.M.M., D.D., A.F. and M.Z. conceived and designed the experiments and edited drafts of the paper; S.D.S. and S.S.S. supervised the research. All authors reviewed and approved the final manuscript.

## Ethics Statement

The study protocol was approved by the ethical committee of the National Institute for Medical Research Development of Iran (ID: 940604).

## Consent

A written informed consent was obtained from all patients.

## Conflicts of Interest

The authors declare no conflicts of interest.

## Supporting information


Data S1.

## Data Availability

The data that support the findings of this study are available from the corresponding author upon reasonable request.
