# Supplementary material for: The Comparison of the Gut Microbiome Composition, Serum Inflammatory Markers and Faecal Short‐Chain Fatty Acids Among Individuals With Type 1 and 2 Diabetes Mellitus With Healthy Controls: A Case–Control Study
Source: Endocrinol Diabetes Metab. 2025 Jun 17;8(4):e70071. doi: 10.1002/edm2.70071 (PMC12171999; doi:10.1002/edm2.70071)
Supplement: Supplementary file 1 — Data S1. [file EDM2-8-e70071-s001.docx]

**Supplementary Table.** Oligonucleotides sequences for the 16S rRNA gene of bacteria of each bacteria

| **Target Bacteria** | **Oligonucleotide sequence** | **Amplicon size (bp)** | **Reference** |
| --- | --- | --- | --- |
| ***Escherichia*** | **F:** CATTGACGTTACCCGCAGAAGAAGC  **R:** CTCTACGAGACTCAAGCTTGC | 190 | Bartosch (2004) [1] |
| ***Prevotella*** | **F:** CACCAAGGCGACGATCA  **R:** GGATAACGCCYGGACCT | 283 | Larsen (2010) [2] |
| ***Lactobacillus*** | **F:** AGCAGTAGGGAATCTTCCA  **R:** CACCGCTACACATGGAG | 341 | Kanno (2009) [3] |
| ***Bifidobacterium*** | **F:** TCGCGTCYGGTGTGAAAG  **R:** CCACATCCAGCRTCCAC | 243 | Rinttila (2004) [4] |
| ***Akkermansia*** | **F:** CAGCACGTGAAGGTGGGGAC  **R:** CCTTGCGGTTGGCTTCAGAT | 329 | Schneeberger (2015) [5] |
| ***Roseburia*** | **F:** TACTGCATTGGAAACTGTCG  **R:** CGGCACCGAAGAGCAAT | 230 | Larsen (2010) [2] |
| ***Faecalibacterium*** | **F:** GGAGGAAGAAGGTCTTCGG  **R:** AATTCCGCCTACCTCTGCACT | 248 | Fitzgerald (2018) [6] |
| ***Bacteroides*** | **F:** GGTGTCGGCTTAAGTGCCAT  **R:** CGGAYGTAAGGGCCGTGC | 140 | Gregory (2015) [7] |

**bp:** Base pairs; **F:** Forward; **R:** Reverse.

**References**

1. Bartosch S, Fite A, Macfarlane GT, McMurdo ME. Characterization of bacterial communities in feces from healthy elderly volunteers and hospitalized elderly patients by using real-time PCR and effects of antibiotic treatment on the fecal microbiota. Applied and environmental microbiology. 2004;70(6):3575-81.

2. Larsen N, Vogensen FK, van den Berg FW, Nielsen DS, Andreasen AS, Pedersen BK, et al. Gut microbiota in human adults with type 2 diabetes differs from non-diabetic adults. PLoS One. 2010;5(2):e9085.

3. Kanno T, Matsuki T, Oka M, Utsunomiya H, Inada K, Magari H, et al. Gastric acid reduction leads to an alteration in lower intestinal microflora. Biochem Biophys Res Commun. 2009;381(4):666-70.

4. Rinttilä T, Kassinen A, Malinen E, Krogius L, Palva A. Development of an extensive set of 16S rDNA-targeted primers for quantification of pathogenic and indigenous bacteria in faecal samples by real-time PCR. J Appl Microbiol. 2004;97(6):1166-77.

5. Schneeberger M, Everard A, Gómez-Valadés AG, Matamoros S, Ramírez S, Delzenne NM, et al. Akkermansia muciniphila inversely correlates with the onset of inflammation, altered adipose tissue metabolism and metabolic disorders during obesity in mice. Scientific Reports. 2015;5(1):16643.

6. Fitzgerald CB, Shkoporov AN, Sutton TDS, Chaplin AV, Velayudhan V, Ross RP, Hill C. Comparative analysis of Faecalibacterium prausnitzii genomes shows a high level of genome plasticity and warrants separation into new species-level taxa. BMC genomics. 2018;19(1):931.

7. Gregory KE, LaPlante RD, Shan G, Kumar DV, Gregas M. Mode of Birth Influences Preterm Infant Intestinal Colonization With Bacteroides Over the Early Neonatal Period. Advances in neonatal care : official journal of the National Association of Neonatal Nurses. 2015;15(6):386-93.
